# Evaluation of the predicted error of the soil moisture retrieval from C-band SAR by comparison against modelled soil moisture estimates over Australia

**DOI:** 10.1016/j.rse.2011.09.031

**Published:** 2012-05-15

**Authors:** Marcela Doubková, Albert I.J.M. Van Dijk, Daniel Sabel, Wolfgang Wagner, Günter Blöschl

**Affiliations:** a Institute of Photogrammetry and Remote Sensing, Vienna University of Technology, 1040 Vienna, Austria; b Water Information R&D Alliance/CSIRO Water for a Healthy Country, G.P.O. Box 1666, Canberra, ACT 2601, Australia; c Institute of Hydraulic Engineering and Water Resources Management and the Centre for Water Resource Systems (CWRS), Vienna University of Technology, Karlsplatz 13/222, A-1040 Vienna, Austria

**Keywords:** Synthetic Aperture Radar (SAR), Soil moisture, ASAR GM, Error evaluation, Australia, Australian Water Resources Assessment System (AWRA)

## Abstract

The Sentinel-1 will carry onboard a C-band radar instrument that will map the European continent once every four days and the global land surface at least once every twelve days with finest 5 × 20 m spatial resolution. The high temporal sampling rate and operational configuration make Sentinel-1 of interest for operational soil moisture monitoring. Currently, updated soil moisture data are made available at 1 km spatial resolution as a demonstration service using Global Mode (GM) measurements from the Advanced Synthetic Aperture Radar (ASAR) onboard ENVISAT. The service demonstrates the potential of the C-band observations to monitor variations in soil moisture. Importantly, a retrieval error estimate is also available; these are needed to assimilate observations into models. The retrieval error is estimated by propagating sensor errors through the retrieval model.

In this work, the existing ASAR GM retrieval error product is evaluated using independent top soil moisture estimates produced by the grid-based landscape hydrological model (AWRA-L) developed within the Australian Water Resources Assessment system (AWRA). The ASAR GM retrieval error estimate, an assumed prior AWRA-L error estimate and the variance in the respective datasets were used to spatially predict the root mean square error (RMSE) and the Pearson's correlation coefficient *R* between the two datasets. These were compared with the RMSE calculated directly from the two datasets. The predicted and computed RMSE showed a very high level of agreement in spatial patterns as well as good quantitative agreement; the RMSE was predicted within accuracy of 4% of saturated soil moisture over 89% of the Australian land mass. Predicted and calculated *R* maps corresponded within accuracy of 10% over 61% of the continent. The strong correspondence between the predicted and calculated RMSE and *R* builds confidence in the retrieval error model and derived ASAR GM error estimates.

The ASAR GM and Sentinel-1 have the same basic physical measurement characteristics, and therefore very similar retrieval error estimation method can be applied. Because of the expected improvements in radiometric resolution of the Sentinel-1 backscatter measurements, soil moisture estimation errors can be expected to be an order of magnitude less than those for ASAR GM. This opens the possibility for operationally available medium resolution soil moisture estimates with very well-specified errors that can be assimilated into hydrological or crop yield models, with potentially large benefits for land-atmosphere fluxes, crop growth, and water balance monitoring and modelling.

## Highlights

► The error of the existing ENVISAT ASAR GM soil moisture product was assessed. ► Independent top soil moisture estimates produced by the AWRA model were used. ► A high quality of the ASAR GM error estimate was demonstrated. ► The identical error model was proposed for the potential Sentinel-1 product.

## 1 Introduction

To support the operational use of Synthetic Aperture Radar (SAR) earth observation systems, the European Space Agency (ESA) is developing Sentinel-1, a constellation of two polar-orbiting C-band radar satellites. Much like its SAR predecessors (Earth Resource Satellite, ENVISAT and RADARSAT) the Sentinel-1 will operate at a medium spatial resolution, but with a greatly improved revisit period. Each of the Sentinel-1 satellites is expected to provide coverage over Europe and Canada once every four days and global coverage in twelve days or less. Given the high temporal sampling and the operational configuration Sentinel-1 is expected to be beneficial for operational monitoring of dynamic processes in hydrology and phenology.

The benefit of a C-band SAR monitoring service in hydrology has already been demonstrated within the scope of the Soil Moisture for Hydrometeorologic Applications (SHARE) project (http://www.ipf.tuwien.ac.at/radar/share/) (Bartsch, 2008; Doubkova et al., 2009). SHARE is one of the ESA's Data User Element (DUE) Tiger Innovator projects. Within the project a soil moisture dataset at medium resolution was retrieved from the Global Mode (GM) of the Advanced Synthetic Aperture Radar (ASAR) onboard ENVISAT (Pathe et al., 2009). Existing coarse resolution soil moisture data from active and passive sensors (Kerr et al., 2010; Naeimi et al., 2009; Njoku et al., 2003; Wagner, 1998; Wagner et al., 1999b) were found beneficial for weather prediction, climate monitoring or flood forecasting (Brocca et al., 2010; Liu et al., 2010) at global to regional scale. It can be anticipated that further applications would become feasible with medium resolution datasets. These include crop yield and soil moisture monitoring over heterogeneous landscapes and river runoff prediction at areas with local precipitation patterns (Komma et al., 2008; Meier et al., 2011; Osborne et al., 2009; Parajka et al., 2009).

The SHARE project demonstrated the potential of C-band observations at high temporal and moderate spatial resolution to monitor variations in soil moisture on a quasi-operational basis (Pathe et al., 2009). Since the start of the project in 2005, the retrieved soil moisture estimates have been requested by more than 80 organisations worldwide, the main application domains being hydrology, agriculture and comparison with other soil moisture datasets. A published validation study demonstrated a good correspondence of ASAR GM soil moisture with in-situ data and airborne SAR systems (Mladenova et al., 2010). Possible applications of the ASAR GM data included bias identification in the precipitation datasets (Milzow et al., 2010) and support for runoff monitoring (Bartsch et al., 2007). It was nevertheless concluded that the usability of the dataset is compromised by the intermittent coverage and poor radiometric resolution of the sensor in global mode (Wagner et al., 2010). Sentinel-1 will improve revisit period and radiometric resolution and so overcome the major limitations of the ASAR GM sensor. Given the otherwise similar sensor characteristics (Attema et al., 2007), the transfer of the ASAR GM soil moisture service to Sentinel-1 seems an obvious opportunity.

A common approach for demonstrating the benefit of satellite-derived data relies on their assimilation into existing models. Assimilation techniques require accurate estimates of observational errors (Liu et al., 2010; Scipal et al., 2008). Pathe et al. (2009) developed an ASAR GM error propagation model. This model predicts the ASAR GM soil moisture error using the Gaussian error propagation scheme.

In this study the ASAR GM soil moisture error estimates produced following Pathe et al. (2009) are evaluated using independent surface soil moisture estimates from the grid-based landscape hydrological model (AWRA-L) developed within the Australian Water Resource Assessment modelling system (AWRA; Van Dijk, 2010). In particular, the RMSE and *R* computed between the satellite and modelled data are predicted using the ASAR GM error estimates and compared to the observed RMSE and *R* between the two soil moisture datasets.

This paper is organised as follows. The theory, methodology and data sections introduce the models used for the RMSE and *R* computation, processing steps and the data. The discussion and result sections present: a) an evaluation of the correlation between the satellite (ASAR GM) and modelled (AWRA-L) soil moisture datasets to determine if these capture the same processes; and b) an assessment of the ASAR GM error estimate using the models for the RMSE and *R* prediction. The implications for a possible future Sentinel-1 soil moisture product are also discussed. Conclusions and future recommendations are summarized in the final section.

## 2 Theory

The RMSE and *R* between two datasets can be calculated directly from the variance and covariance statistics; these will be refereed to as the observed values. In addition, if the error (*ε*) and variance (*σ*^*2*^) of the respective datasets are known, the RMSE and *R* values can be predicted; these will be refereed as the predicted values.

Although the AWRA-L and ASAR GM soil moisture estimates are assumed to represent the same phenomenon, they are expressed in different units. This may induce a bias in the mean and dynamic range that opposes the model assumption (Dee & Todling, 2000). To correct for possible biases the AWRA-L dataset was adjusted to the ASAR GM dataset using Cumulative Distribution Function (CDF) matching techniques.

Because the goal of this study was to evaluate the quality of the existing satellite error estimate, the modelled data were scaled with respect to the satellite data (for data assimilation studies, an inverse approach is more logical; Reichle & Koster, 2004). The transformation of the AWRA-L soil moisture estimates used in this study is a CDF matching technique simplified to a linear transformation that effectively removes the differences in the first two moments (i.e. mean and variance):

(1) $$\theta_{M}=\frac{\left(\theta_{M,or}-{\bar{\theta }}_{M,or}\right)}{stdev_{M,or}}*stdev_{S}+{\bar{\theta }}_{S}\text{,}$$

where *θ* represents the soil moisture observations, and *stdev* and ${\bar{\theta }}_{}$ the temporal standard deviation and the temporal mean of these observations, respectively. The subscript *S* and *M* symbolize the satellite and modelled dataset, respectively. Finally, the subscript *or* indicates the original dataset before normalisation. In all subsequent computations the normalized AWRA-L soil moisture estimates (*θ*_*M*_) were used.

### 2.1 Observed RMSE and R

The RMSE is a straightforward measure of estimation accuracy between two datasets. The RMSE_a_ between modelled and satellite-derived soil moisture can be defined through the variance of residual errors. If *θ*_*S*_ is the satellite-derived soil moisture and *θ*_*M*_ the normalised modelled soil moisture then the RMSE_a_ is defined as

(2) $$RMSE_{a}=\sqrt{\langle {\left(\theta_{M}-\theta_{S}\right)}^{2}\rangle }\text{,}$$

where the angle brackets represent the mean over time.

The RMSE_a_ in combination with the variances of the satellite and modelled data can be used to calculate the correlation coefficient *R*_*a*_ (Barnston, 1992; Murphy, 1995):

(3) $$R_{a}=\frac{stdev_{M}^{2}+stdev_{S}^{2}-RMSE_{a}^{2}}{2stdev_{M}stdev_{S}}\text{,}$$

where *stdev*_*M*_ and *stdev*_*S*_ stand for the temporal standard deviation of the normalized modelled and satellite derived soil moisture, respectively.

### 2.2 Predicted RMSE and R

The RMSE can be predicted from the error characteristics of the satellite (*ε*_*S*_) and the modelled (*ε*_*M*_) data using error propagation model of (Taylor, 1997):

(4) $$RMSE_{b}=\sqrt{{\langle \varepsilon_{M}\rangle }^{2}+{\langle \varepsilon_{S}\rangle }^{2}}\text{.}$$

The assumptions of the error propagation model are that the respective error characteristics are independent and follow a Gaussian normal distribution. The assumption on error characteristics is realistic as the main input data to the AWRA-L, daily precipitation, incoming shortwave radiation and temperature, are independent of the ASAR GM backscatter. Moreover, the Gaussian normal distribution of the error estimates can be anticipated given the normal distribution of the AWRA-L inputs and the ASAR GM backscatter.

By substituting Eq. (4) into Eq. (3) the correlation coefficient can be predicted:

(5) $$R_{b}\approx \frac{stdev_{M}^{2}+stdev_{S}^{2}-\langle \varepsilon_{M}^{2}\rangle -\langle \varepsilon_{S}^{2}\rangle }{2stdev_{M}stdev_{S}}\text{.}$$

It should be noted that due to the prior normalisation of the data, the RMSE_b_ and *R*_*b*_ metrics capture the correspondence of two datasets in their dynamics regardless of biases in mean or variance.

### 2.3 ASAR GM soil moisture error (ε_S_)

The maximum ASAR GM soil moisture error (*ε*_*S*_) was estimated following Pathe et al. (2009). The method uses a Guassian error propagation scheme to propagate the backscatter noise and retrieval model parameter uncertainty according to:

(6) $$\varepsilon_{S}=\sqrt{{\left(\frac{1.2}{S}\right)}^{2}+{\left(\frac{\beta }{S}\right)}^{2}+0.01}\text{.}$$

The ASAR GM product was derived using the change detection model (Pathe et al., 2009) defined as:

(7) $$\theta_{S}=\frac{\sigma^{0}(\Theta ,t)-\sigma_{\mathrm{dry}}^{0}(30)-\beta (\Theta -30)}{S}\text{,}$$

where *σ*^*o*^_*dry*_ (30), *β* and *S* are considered constant in time and represent respectively the dry reference at medium local incidence angle 30°, the slope, and the sensitivity of the ASAR GM backscatter to soil moisture. The slope quantifies the dependence of sigma nought on the local incidence angle. The *σ*^o^(Θ,t) stands for the backscatter values at an local incidence angle *Θ* in time *t*.

The dry reference has been derived according to Pathe et al. (2009) using historical ERS scatterometer soil moisture archive. The application of the external soil moisture dataset is possible given the identical frequency and penetration depth of the ASAR GM 1-km and ERS sensors. While systematic bias may be introduced due to the difference in the noise and the time coverage of the ERS and ASAR GM data (Pathe et al., 2009) this is removed during the data normalisation.

### 2.4 ASAR GM soil moisture

Data from the multiple modes of the side-looking Synthetic Aperture Radar (SAR) onboard ENVISAT are available since December 2004. The ASAR Global Monitoring Mode (GM) is activated by default when no data from other modes are requested. The ASAR GM 1 km resolution sensor thus offers higher temporal sampling over certain regions when compared to other modes and is suitable for monitoring of dynamical processes such as soil moisture (Pathe et al., 2009) or inundation (Bartsch et al., 2009).

**Fig. 1:**
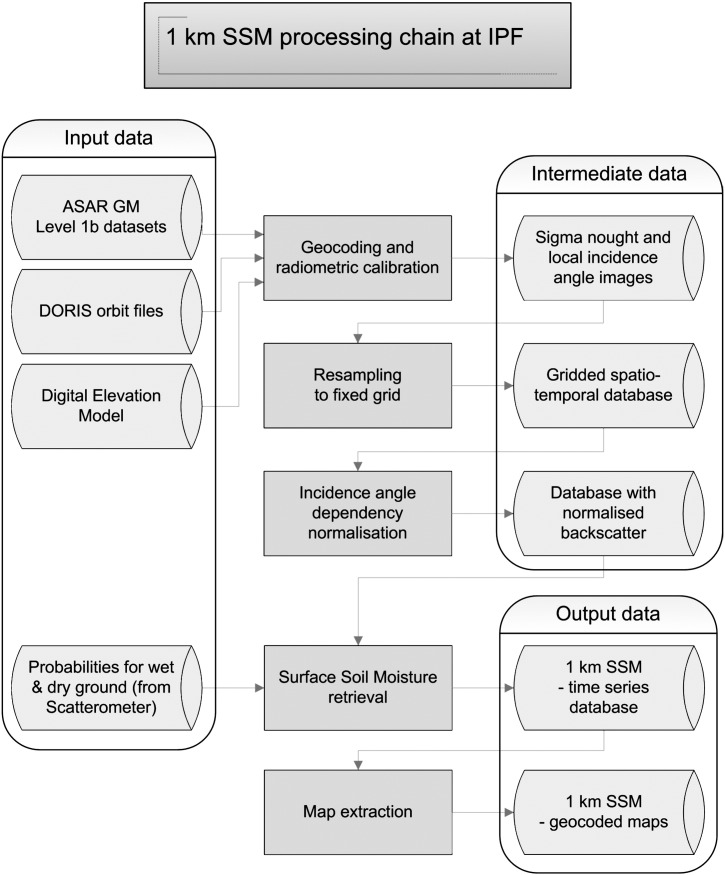
The processing chain of the ASAR GM data at the TU WIEN (Sabel et al., 2010).

The algorithm used to retrieve soil moisture from the ASAR GM observations was derived from the soil moisture algorithm for the Earth Resource Satellite (ERS) scatterometer (Wagner et al., 2007). The approach is based on a change detection method and assumes a) sufficiently long time series to cover a full range of soil moisture values from wilting point to saturation (Pathe et al., 2009; Wagner et al., 1999a, 1999b) and b) variations in soil moisture to be tracked by temporal change in backscatter (Moran et al., 2006; Pathe et al., 2009). Exceptions to the rule are regions covered with dense vegetation. For the soil moisture product generation a processing chain has been setup at the Vienna University of Technology (TU WIEN) (Sabel et al., 2010). The processing consists of several steps including geocoding, radiometric correction, resampling, normalisation and soil moisture retrieval (Fig. 1). The expected depth represented by the ASAR GM soil moisture product is less than 5 cm. Importantly, over 7000 ASAR GM scenes over Australia were used in this study.

The ASAR GM estimates were evaluated against in-situ measurements and retrievals from other satellites (Mladenova et al., 2010; Pathe et al., 2009). This demonstrated the potential of the dataset to resolve spatial details that are not resolved in the ERS scatterometer measurements, while still retaining the basic capability to capture drying and wetting trends over large areas.

**Fig. 2:**
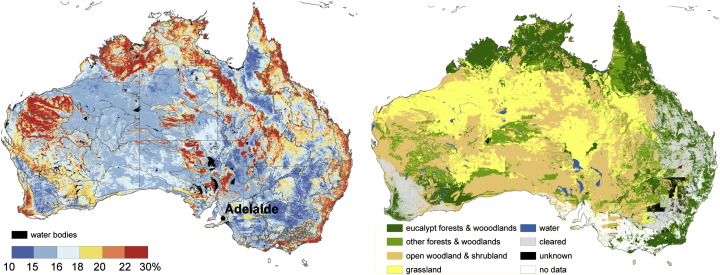
The maximum surface soil moisture retrieval error SM_max_ for Australia calculated using error propagation model (Pathe et al., 2009) (left) and the present major vegetation groups (Australian Government Department of the Environment and Water Resources, 2005) (right). The SM_max_ is overlaid with the Interim Biogeographic Regionalization dataset for Australia — IBRA (Thackway and Creswell, 1995).

The maximum ASAR GM soil moisture error *ε*_*S*_ was computed according to Eq. (6). The error map shown in Fig. 2 (left) for Australia strongly coincides with spatial patterns of a combination of vegetation type (Fig. 2, right) and landscape geomorphology (Van Dijk & Warren, 2010). In particular, the error is less (< 18%) for herbaceous and shrub vegetation classes and greater for forested areas and areas covered with rock outcrops in western, northern, and eastern coastal Australia. Correspondence of the ASAR GM error with the bioregions of the Interim Biogeographic Regionalisation (IBRA; Thackway and Creswell, 1995) is also evident (Fig. 2). The IBRA mapping combines attributes of climate, geomorphology, landform and lithology.

### 2.5 AWRA-L soil moisture

The AWRA (Van Dijk, 2010) consists of a selection of models that estimate all water balance terms associated with the vegetation, soil, groundwater and surface water balance. The models operate at moderate to high resolution across the Australian continent. With a view to assimilate satellite-derived soil moisture observations, a gridded landscape hydrology model (AWRA-L) was built as a sub-model of the AWRA system (Van Dijk, 2010) to explicitly model soil surface moisture dynamics.

The AWRA-L landscape hydrological model estimates the soil water balance at a daily step for four different layers: the surface top soil, the shallow root zone, the deep root zone and the saturated ground water store. These are defined by their extractable water storage capacity that depends on the pore size distribution, soil porosity and storage capacity. The conceptual differences are that the surface soil layer loses water through direct evaporation; the shallow root zone is accessed by all vegetation; and the deep root zone can be accessed by deep-rooted (usually perennial) vegetation only. Top soil moisture storage *S*_*0*_ (mm) at time step *t* is estimated by:

(8) $$S_{0}\left(t+1\right)=S_{0}\left(t\right)+I\left(t\right)-E_{S}\left(t\right)-D_{0}\left(t\right)\text{,}$$

where *I* is infiltration, *E*_*S*_ soil evaporation and *D*_*0*_ top soil drainage (all in mm/d). The model is based on the energy and mass balance equations and uses empirical relationships to estimate the fluxes. The evaporation part of the model is critically important for the soil moisture estimates. It accounts for rainfall interception evaporation, soil evaporation and transpiration; the latter two using the Penman–Monteith equation model. AWRA-L parameters were derived from the literature and analysis of streamflow data from several hundred Australian catchments. Full details on the model and its implementation can be found in Van Dijk (2010).

A soil moisture estimate comparable to the relative satellite-derived soil moisture product can be calculated as:

(9) $$\theta_{M}=\frac{S_{0}}{S_{0.FC}}$$

where *S*_*0,FC*_ is the top soil water storage capacity between field capacity and the point at which evaporation ceases (wilting point). *S*_*0FC*_ was estimated at 30 mm across the continent, corresponding to 0-z cm of the top soil layer, where *z* ranges between 5 and 10 cm. While this differs from the depth represented by the ASAR GM (< 5 cm), high correlations are expected between the two layers due to their hydraulic coupling. Potentially, portion of the bias removed during the normalisation may also be induced by the difference in the depth represented by the ASAR GM and AWRA-L soil moisture products. The AWRA-L soil moisture is estimated at 0.05° spatial resolution and daily time step. Errors in AWRA-L soil moisture estimates arise from a) the model structure, b) the model parameters, and c) the data used to force the model (Van Dijk & Warren, 2010). The errors in the model structure are caused by the inevitable simplification of the processes regulating soil moisture dynamics. The errors of the model parameters are dominated by the inability to obtain optimal spatial parameter sets across large areas. The errors in input originate mainly in station measurement and interpolation. Precipitation errors in particular have been shown to strongly affect the agreement in satellite and model soil moisture (Crow et al., 2009; Draper et al., 2009; McCabe et al., 2008).

## 3 Methodology

The spatial aggregation was recommended to reduce the noise of the ASAR GM soil moisture when used in applied studies (Pathe et al., 2009). Given the nature of this publication, assessing the error structure of the ASAR GM soil moisture product, all analyses were performed at the original 1 km resolution. This avoids lost of information and problems with result interpretation that may arise due to the data aggregation. The AWRA-L 0.05° resolution estimates were oversampled to the ASAR GM 1 km grid (Sabel et al., 2010) using the nearest neighbour technique.

First, the ASAR GM and AWRA-L soil moisture estimates are assessed in order to determine if these capture the same processes. For this purpose the Pearson's correlation coefficient *R* is studied.

Secondly, the quality of the ASAR GM error estimate is evaluated. In particular, the ability of the ASAR GM error to predict the RMSE_b_ between the satellite-derived and modelled soil moisture is studied. A model is used that relates the RMSE_b_ to the individual errors of each dataset according to Eq. (4). The RMSE_a_ is calculated from the observations according to Eq. (2). Given the independence of the two methods, a high correspondence between the RMSE_a_ and RMSE_b_ suggests a high quality of the RMSE_b_ model and the individual error estimates.

The RMSE_b_ computation according to Eq. (4) is complicated by the limited knowledge of the modelled dataset error *ε*_*M*_ (Van Dijk & Warren, 2010). In a first approximation, *ε*_*M*_ was assumed to be constant and equal to 15% of the soil moisture content at field capacity (30 mm). Given the top soil water storage of 30 mm corresponding to ca. 5 to 10 cm of the top soil layer, *ε*_*M*_ of 15% accounts for an error of 4.5–9 mm what corresponds to 0.045–0.09 m^3^/m^3^. This seems as a realistic error estimate for an uncalibrated model (Choi et al., 2002; Crawford et al., 2000). The assumption on a constant behaviour of *ε*_*M*_ is unlikely to be accurate, either spatially or temporally, but was necessary due to the lack of independent spatial estimates other than ASAR GM. Where possible the difference between the RMSE_a_ and RMSE_b_ is qualitatively assigned to the satellite or to the modelled data.

The quality of the *R*_*b*_ is assessed using the observed *R*_*a*_ computed from the observations with Eq. (3). The aim of the estimation is twofold. First, it provides a fast assessment of *R* with only limited knowledge of the *stdev* and error of the modelled and satellite datasets. Secondly, it evaluates the quality of the individual error estimates by evaluating their ability to predict the *R*_*b.*_ Being insensitive to any retrieval bias, the knowledge of the *R* metric is often needed for the retrieval assimilation into a model.

Two simplifications in the estimation of the *R*_*b*_ needed to be made similar to those implemented within the estimated RMSE_*b*_: a) a constant error of 15% was assumed in the modelled data; and b) a constant variance of 15% was assumed in the modelled data. The realism of these assumptions is evaluated by comparison of the *R*_*b*_ and *R*_*a*_ and the potential source of the *R* differences is addressed.

## 4 Results and discussion

### 4.1 Correlation between ASAR GM and AWRA-L soil moisture

The ASAR GM and AWRA-L soil moisture estimates are assessed for similarity in soil moisture dynamics. The Pearson's correlation coefficient and the RMSE_a_ are computed over the entire continent for the period 2005–2009 (Figs. 4 and 5).

**Fig. 3:**
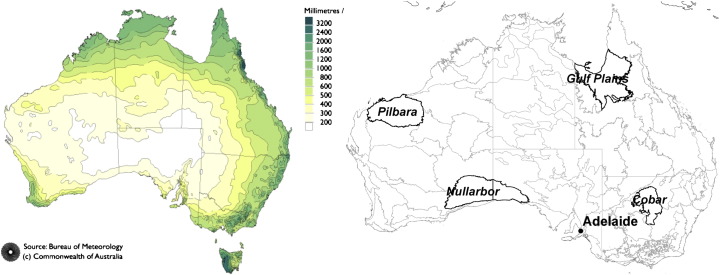
Mean Annual Precipitation (source: Bureau of Meteorology) (left) and the Interim Biogeographic Regionalisation dataset for Australia (IBRA) with four selected regions (right) (Thackway & Creswell, 1995).

**Fig. 4:**
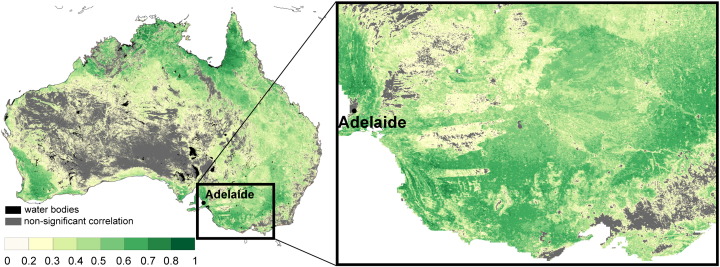
The Pearson's correlation coefficient between the ASAR GM and the AWRA-L soil moisture data over Australia. The grey areas in the correlation map display the non-significant correlation values (p = 0.05%).

An overall high agreement between the ASAR GM and the AWRA-L soil moisture observations is demonstrated in Fig. 4. Significant correlations were found over 72% of the continent. High correlation values (*R* > 0.6) dominate in southwest, southeast and northern Australia. Areas with high correlations in southwest and southeast of Australia correspond to dry land cropping regions. Regions with high correlations can be characterised by high mean annual precipitation (Fig. 3) and vegetation dominated by herbaceous plants (Fig. 2). This agrees with a priori expectation based on the physics of radiation transfer: the sparse vegetation allows for a good penetration of C-band signal and increases so the ASAR GM sensitivity to soil moisture. Australia's wetter regions generally also have a greater density of precipitation gauging stations, which may enhance the quality of the AWRA-L rainfall forcing and reduce error in soil moisture estimates.

Insignificant correlations were found over portions of central arid, north-western and eastern coastal Australia and correspond well to areas with high ASAR GM error (Fig. 2). The potential reasons for lesser agreement in dry areas are the low mean and variance of mean annual precipitation causing low variance in soil moisture and the lesser quality of the rainfall data and hence the model estimates. The signal-to-noise ratio over arid regions is expected to be minimal considering the low mean and variance of soil moisture data and the poor radiometric resolution of the ASAR GM (~ 1.2 dB) data. The low correlation in eastern coastal areas may be explained by the limited ASAR GM sensitivity to soil moisture due to dense vegetation, heterogeneous relief and widespread urban development.

The correlation coefficient between ASAR GM and AWRA-L soil moisture seems to be dependent also at medium scale representing dependency on different vegetation forms. For instance, correlation is low along major rivers due to the presence of floodplain forests (Fig. 4, right). Similarly, remnant native mallee bush land areas (linear shapes east of Adelaide) show lower *R* compared to the surrounding agricultural (Fig. 4, right).

The two datasets are fully independent as the main input data to the AWRA-L are independent of the ASAR GM backscatter. Given this independence, the high correlations support the notion that they represent the same phenomenon.

### 4.2 RMSE model performance

**Fig. 5:**
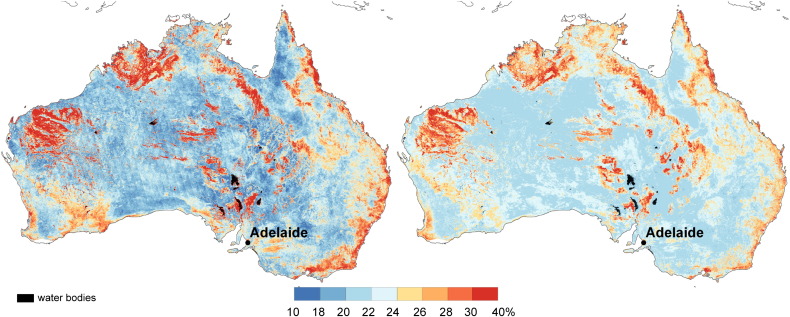
The maps represent the RMSE_a_ computed from the observations (left) and the RMSE_b_ predicted (Eq. 4) (right).

The RMSE_a_ and RMSE_b_ maps are displayed in Fig. 5. An overall very high agreement of spatial patterns is evident. The areas with high values (> 30%) coincide in both maps and cover regions associated with steep slopes and rock outcrops (e.g. rock outcrops in northern and Western Australia). Errors above 30% are also encountered along the eastern coast. The low values (< 24%) coincide in both maps and often correspond to areas with high *R* (> 0.6) demonstrated in Fig. 4. These are alluvial, topographically uniform areas, or areas covered with herbaceous growth (e.g. alluvial region in the Gulf Plains in northern Queensland or the Nullarbor bioregion in southern Australia displayed in Fig. 3) that exhibit relatively high mean annual precipitation. Low values also dominate in central arid regions with only limited mean annual precipitation (Fig. 3).

The high values over rock outcrops in central, northern and Western Australia can be attributed to the ASAR GM observational errors. These originate in diffuse scattering from very rough areas or in foreshortening effects in steep slopes. The latter is not always corrected during geometric and radiometric correction due to the limitations of the DEM. The high values in eastern Australia may be associated with dense vegetation that lowers sensitivity of the C-band backscatter to soil moisture. Similar findings demonstrating the sensitivity of the RMSE_a_ to the topographical and geomorphological medium scale features were documented by Van Dijk and Warren (2010).

The match between the both RMSE_a_ and RMSE_b_ maps and growth forms (Fig. 2) is pronounced in the southwestern and eastern Australia. Especially the crossover between herbaceous growing forms and shrubs is evident. As example may serve the so-called Menzies Line in southwest Australia dividing herbaceous vegetation on cleared land from native shrubland; the sharp divisions between cropping and grazing land east of Adelaide, and the Cobar bioregion in eastern Australia that is dominated by small trees (Fig. 2). These regions are easily detectable due to their specific land cover forms and also due to the specific soil and explicit base rock type (Van Dijk & Warren, 2010).

**Fig. 6:**
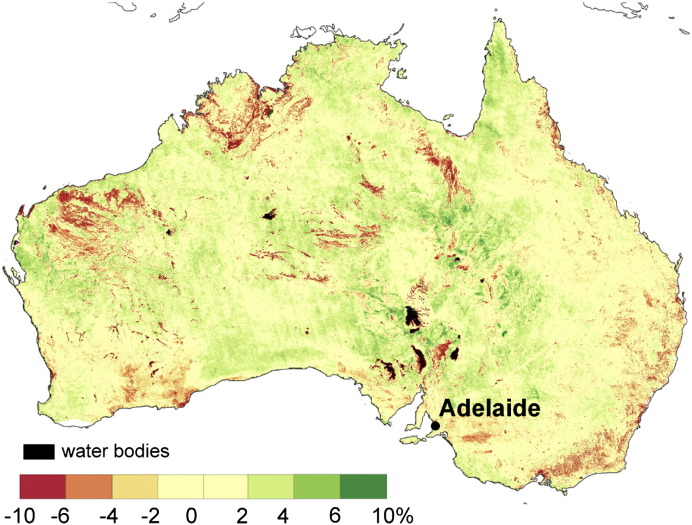
The difference between the RMSE_b_ and the RMSE_a_ computed from the observations.

The difference between the RMSE_b_ and RMSE_a_ is displayed in Fig. 6. The RMSE_b_ corresponded to the RMSE_a_ wthin ± 4% of saturated soil moisture over 89% of the land mass. The remaining 11% coincides mainly with rock outcrops, salt pans and densely vegetated areas (Fig. 2).

The RMSE_b_ underestimates the RMSE_a_ over areas with steep slopes and rock outrcrop areas in central, western and northwestern Australia (red colours in Fig. 6). This underestimation may originate from the ASAR GM (Fig. 2) as well as from the AWRA-L soil moisture error. The AWRA-L soil moisture estimates are likely to be poor where the surface is dominated by hard rock outcrops or salt lakes, as the model parameterization does not explicitly consider these features. Nevertheless, the AWRA-L errors are expected to be mainly related to the errors in rainfall forcing (Van Dijk & Warren, 2010), and thus, correspond to relatively large scale patterns. The RMSE_b_ is also lower than the RMSE_a_ in eastern coastal Australia. The reverse performance is found over large portions of central and Western Australia (green colours in Fig. 6). Given the limited mean annual precipitation (Fig. 3) over these regions it is suggested that the error estimate of the AWRA-L model may be lower than the anticipated 15% (0.045–0.09 m^3^/m^3^).

Overall, the results demonstrate a very high agreement between the RMSE_a_ and RMSE_b_ estimated according to Eq. (2). Given the independence of the two methods the high correspondence of the RMSE_a_ and RMSE_b_ maps suggests a good accuracy of the error model and the derived ASAR GM error estimate *ε*_*S*_.

### 4.3 R model performance

**Fig. 7:**
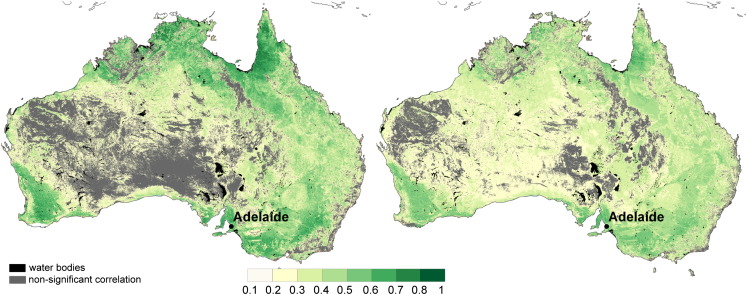
The Pearson's correlation coefficient between ASAR GM and AWRA-L soil moisture. The maps represent R_a_ calculated from the ASAR GM and the AWRA-L observations (left) and the R_b_ (Eq. 5) (right). The grey areas display the non-significant correlation values.

The comparison of the correlation coefficient *R*_*a*_ computed from the observations with the *R*_*b*_ computed according to Eq. (5) is presented in Fig. 7. Despite the simplifying assumptions on uniformity of the AWRA-L error estimate and standard deviation an overall high correspondence is demonstrated at large and medium scale. The correspondence of the large scale patterns is most likely related to atmospherical forcing, mainly to the patterns of mean annual precipitation. The super imposed medium scale patterns are introduced mainly by geometrical properties of vegetation or soil surface (i.e. the bioregion Cobar, wheat belt regions, floodplain vegetation in southeast Australia or patterns of native bush land east of Adelaide).

While the relative patterns of the correlation coefficient correspond well, the absolute values differ. In particular, the *R*_*b*_ is too low over the majority of the continent. It is anticipated that the discrepancies in the *R* are driven by the assumed uniformity of the AWRA-L soil moisture error and variance according to Eq. (5).

The corresponding patterns in Fig. 7 demonstrate the ability of the model to provide a rough estimate of the *R*. The high *R* depicts areas where the ASAR GM and independent soil moisture estimates capture the same processes.

### 4.4 Towards Sentinel-1

Given the similar characteristics of the ASAR GM and Sentinel-1 sensors it is anticipated that the error propagation model can be applied to a potential soil moisture product retrieved from Sentinel-1. Nevertheless, the influence of surface features such as vegetation and roughness at the Sentinel-1 scale needs to be carefully considered. The impact of these effects may require modifications to both the retrieval algorithm and the error model. In addition, the demonstrated modelling difficulties of soil moisture at fine scales (Thoma et al., 2008) suggest that averaging and filtering of the raw Sentinel-1 data to a regional scale (i.e. 0.5 km or 1 km) may be beneficial. The question arises how the effects of surface features will propagate to this scale and if they will be detectable at all. A detailed discussion on the Sentinel-1 algorithm and error model is beyond the scope of this paper, but anticipated modifications are likely to include:

• The improved revisit period may improve the estimation of the individual model parameters.
• The final error is expected to improve by an order of magnitude due to a) the improved radiometric resolution of the Sentinel-1 backscatter measurements (0.128 dB) (Snoeij et al., 2010) comparable to ASAR GM (1.2 dB) and b) the averaging and filtering of the raw data to regional scale (i.e. 0.5 km or 1 km)
• Additional parameters may be necessary in the Sentinel-1 error model that account for the effects of vegetation and surface roughness. Their actual contribution to the final error estimate is linked to the spatial and radiometric resolution of the final Sentinel-1 soil moisture product.

A medium resolution operational soil moisture product with a well-specified error behaviour is proposed. While data assimilation of the ASAR GM soil moisture estimates may be currently restricted by its poor radiometric resolution, the proposed soil moisture product from Sentinel-1 has the potential to be of great benefit for flux exchange, crop growth, and water balance modelling.

## 5 Conclusion

The propagated error of the existing ENVISAT ASAR GM soil moisture product was assessed using independent surface soil moisture modelled by the grid-based landscape hydrological model (AWRA-L) developed within the Australian Water Resource Assessment (AWRA) system.

First, the correspondence of the ASAR GM and AWRA-L estimates was analysed. Given the independence of the two retrieval approaches the high correlation values suggest that the soil moisture estimates capture identical processes. Further, the quality of the ASAR GM error estimate was evaluated by studying its ability to estimate the *R*_*a*_ and RMSE_a_ observed between the satellite and modelled soil moisture. The estimation model relates the RMSE_b_ and *R*_*b*_ to the error characteristics of both datasets. Estimated measures were evaluated against the measures computed from the modelled and satellite observations. This is possible given the independence of the two estimation methods. The high correspondence of the a) RMSE_a_ and RMSE_b_ and b) *R*_*a*_ and *R*_*b*_ maps demonstrated a high quality of the derived models and the ASAR GM error estimate *ε*_*S*_.

The possible reasons for discrepancies in the estimated statistical measures were assessed. These occurred at large ( >  25 km) as well as at medium ( < 1 km) scales. It cannot be clearly distinguished whether the differences in the RMSE and *R* originate from the errors of the AWRA-L model estimate or from the error of the satellite estimate. The medium scale differences are expected to be introduced by the underestimation of the satellite error over vegetated forms or rock outcrops. The large scale differences may originate from an overestimation of the AWRA-L model error in arid regions.

The presented validation approach for the satellite error estimate is transferable to other modelled and satellite-derived data provided that the respective errors are independent. It is expected that an improvement to the validation approach can be achieved with a better understanding of the modelled data error.

It is suggested that an error propagation model similar to that introduced for ASAR GM can be applied for the Sentinel-1 soil moisture error retrieval. It is expected that the retrieval error will be in an order of magnitude lower when compared to the ASAR GM. The suggested operationally available medium resolution soil moisture from Sentinel-1 with a well-specified error is likely to carry strong benefits for modelling and monitoring of land surface-atmosphere fluxes, crop growth and the water balance.
